# Long non-coding RNA expression profiles predict metastasis in lymph node-negative breast cancer independently of traditional prognostic markers

**DOI:** 10.1186/s13058-015-0557-4

**Published:** 2015-04-11

**Authors:** Kristina P Sørensen, Mads Thomassen, Qihua Tan, Martin Bak, Søren Cold, Mark Burton, Martin J Larsen, Torben A Kruse

**Affiliations:** Department of Clinical Genetics, Odense University Hospital, Sdr. Boulevard 29, 5000 Odense C, Denmark; Human Genetics, Clinical Institute, University of Southern Denmark, Sdr. Boulevard 29, 5000 Odense C, Denmark; Epidemiology, Institute of Public Health, University of Southern Denmark, J.B. Winsløvs Vej 9, 5000 Odense C, Denmark; Department of Pathology, Odense University Hospital, J.B. Winsløvs Vej 15, 5000 Odense C, Denmark; Department of Oncology, Odense University Hospital, Sdr. Boulevard 29, 5000 Odense C, Denmark

## Abstract

**Introduction:**

Patients with clinically and pathologically similar breast tumors often have very different outcomes and treatment responses. Current prognostic markers allocate the majority of breast cancer patients to the high-risk group, yielding high sensitivities in expense of specificities below 20%, leading to considerable overtreatment, especially in lymph node-negative patients. Seventy percent would be cured by surgery and radiotherapy alone in this group. Thus, precise and early indicators of metastasis are highly desirable to reduce overtreatment. Previous prognostic RNA-profiling studies have only focused on the protein-coding part of the genome, however the human genome contains thousands of long non-coding RNAs (lncRNAs) and this unexplored field possesses large potential for identification of novel prognostic markers.

**Methods:**

We evaluated lncRNA microarray data from 164 primary breast tumors from adjuvant naïve patients with a mean follow-up of 18 years. Eighty two patients who developed detectable distant metastasis were compared to 82 patients where no metastases were diagnosed. For validation, we determined the prognostic value of the lncRNA profiles by comparing the ability of the profiles to predict metastasis in two additional, previously-published, cohorts.

**Results:**

We showed that lncRNA profiles could distinguish metastatic patients from non-metastatic patients with sensitivities above 90% and specificities of 64-65%. Furthermore; classifications were independent of traditional prognostic markers and time to metastasis.

**Conclusions:**

To our knowledge, this is the first study investigating the prognostic potential of lncRNA profiles. Our study suggest that lncRNA profiles provide additional prognostic information and may contribute to the identification of early breast cancer patients eligible for adjuvant therapy, as well as early breast cancer patients that could avoid unnecessary systemic adjuvant therapy. This study emphasizes the potential role of lncRNAs in breast cancer prognosis.

**Electronic supplementary material:**

The online version of this article (doi:10.1186/s13058-015-0557-4) contains supplementary material, which is available to authorized users.

## Introduction

Breast cancer is the most common cancer in women, affecting more than 10% of women in Western countries. In Western countries, it is the leading cause of death among women below the age of 50 years. The majority of all breast cancer patients are diagnosed as having a high risk of recurrence and are therefore offered adjuvant systemic treatment. This prediction of risk based on clinical and pathological criteria is, however, far from optimal, and considerable overtreatment occurs, especially in the lymph node-negative group of patients. It is of clinical interest to identify biomarkers that could improve prognostic predictions.

Researchers have developed microarray-based gene expression profiles from frozen tumors from patients with good or poor prognosis and the metastasis risk has been predicted by gene expression of the primary tumor [1-4]. Gene expression analyses have also revealed that estrogen receptor (ER)-positive and ER-negative breast cancers are molecularly distinct diseases [5,6] and stratification may be necessary when making prognostic gene signatures [7].

During the past decade, advances in biotechnology such as RNA sequencing have indicated that the majority of the genome is transcribed into non-coding RNA [8]. Increasing knowledge and identification of long non-coding RNAs (lncRNAs) are emerging. lncRNAs are RNA molecules that are longer than 200 nucleotides, having no obvious protein-coding capacity [9,10]. In general, lncRNAs show lower expression and are more tissue-specific compared to protein-coding genes [10].

Different catalogs of several thousand human lncRNAs have been generated from RNA-sequencing data [10,11]. However, only a small number of lncRNAs have been functionally characterized in detail, although studies have associated several lncRNAs with a broad spectrum of biological mechanisms [12]. Generally, lncRNAs are linked to diverse gene-regulatory roles such as chromosome dosage compensation, imprinting, epigenetic regulation, cell-cycle control, nuclear and cytoplasmic trafficking, transcription, translation, splicing, and cell differentiation, et cetera. [13,14]. Most importantly, aberrant expression of lncRNAs is linked to several disease states, including cancer [13,15].

A few studies have associated certain lncRNAs with poor outcome and disease progression in different types of cancer: high *HOTAIR* expression was found in several types of cancer, including breast and colorectal cancer [16-18], overexpression of *PCAT-1* has been observed in prostate cancer [19] and overexpression of *MALAT-1* has been observed in several types of cancer [15], including early-stage non small-cell lung cancer [20].

In this study, we explored whether lncRNAs could predict the clinical outcome in lymph node-negative adjuvant-naïve breast cancer patients and provide independent prognostic information. We selected 82 primary tumors from patients who subsequently developed distant metastasis and pair-matched them to 82 primary tumors from patients who remained metastasis-free. We analyzed the expression of lncRNAs, performed classification and identified lncRNAs that predicted metastasizing primary tumors and non-metastasizing primary tumors independent of classic prognostic markers and with high accuracy.

## Methods

### Patients’ samples

We selected frozen tumor biopsies from lymph node-negative patients who were diagnosed from 1980 to 2003 on the island of Funen, Denmark. All patients underwent surgery for primary breast cancer, but none of the patients received systemic adjuvant therapy. All tumors were ≤5 cm in diameter and were snap-frozen and stored at −80°C. Pathological examination of the samples was performed at the Department of Pathology at Odense University Hospital, and all samples contained >50% tumor cells. We selected biopsies from 82 patients who developed detectable distant metastasis within a range of 0 to 15 years and 82 biopsies from patients with no metastases diagnosed for at least 8 years or longer (one patient did not fulfill this criterion) (mean follow up 18.1 years) and matched them pair wise according to the following criteria: tumor type, year of surgery (range 1980 to 2003), tumor size (range 0.6 to 5 cm), age (range 33 to 88 years), receptor status, and histological grade (grade 1 to 3, or grade not available) (Table 1). We chose the paired design to avoid classifications dependent on traditional prognostic markers. By choosing this design, we also increased the study power by enriching for informative clinical endpoints compared with a cohort study.

**Table 1:** **Patient and tumor characteristics**

	**Metastasizing tumors**	**Non-metastasizing tumors**
**Age at diagnosis (range 33 to 88 years)**		
≤50 years	16	12
>50 years	66	70
**Tumor size**		
≤2 cm	35	36
2 to 5 cm	47	45
Not available*		1
**Estrogen receptor status**		
Positive	58	62
Negative	24	20
**Tumor type**		
Invasive ductal carcinoma	64	67
Invasive lobular carcinoma	9	9
Mucinous carcinoma	2	2
Papillary carcinoma	3	2
Carcinoma with metaplasia	2	2
Not available*	2	
**Histologic grade**		
1 (good)	12	15
2 (intermediate)	29	25
3 (poor)	23	26
Not available*	18	16
**Median year of surgery (range 1980 to 2003)**	1993	1994
**Mean time to metastasis (months)**	48.4	-
**Mean follow up (months)**	-	217
**Vital status (number of patients alive April 2013)**	5	57

Results are presented as number of patients unless stated otherwise. *Not available in the Danish Breast Cancer Cooperative Group (DBCG) database.

Of the 82 patients who were diagnosed with metastasis, 16 had regional metastasis, while the remaining had distant metastasis. We extracted all clinicopathological features, including follow-up information, from the Danish Breast Cancer Cooperative Group (DBCG) database, the Funen pathology database, or the nationwide pathology database. Patients who died from any cause other than breast cancer were censored at the time of death. There was no loss to follow up or censored observations. The study was approved by the Danish National Committee on Health Research (S-VF-20020142). The study was retrospective and we did not obtain informed consent from the patients involved in the study as approved by the Ethical Committee.

### Microarray analysis and re-annotation

Total RNA was isolated from the freshly frozen primary breast tumor biopsies and processed as previously described [18]. We used a modified standard design of the SurePrint G3 Human GE 8x60k oligonucleotide slides (G4102A) provided by Agilent Technologies (Santa Clara, USA) for gene expression analysis. We kept the matched sample pairs together during all steps of RNA extraction, amplification, hybridization, and gene expression analysis. Microarray data have been deposited to the Gene Expression Omnibus [GSE48408].

We matched the chromosomal positions of the probes in the annotation file from Agilent to the chromosomal positions of lncRNAs in the Human RNA catalog from GENCODE version 16 [21] to select the probes covering lncRNAs as previously described [22].

### Statistical methods

#### Classification and feature selection

For classification, a support vector machine (SVM) was applied with sigmoid kernel. The models were developed using a threshold providing at least 90% sensitivity, and maximizing the specificity, assessed using leave one pair out cross-validation (LOPOCV). Briefly, in this procedure a single pair of matched samples served as test samples and the remaining samples as a training set. This was repeated until all pairs had been left out once and the accuracy of the classifier was determined by the correctly classified samples. The LOOCV procedure provides an unbiased performance estimate and is the optimal method in small datasets [23,24].

In the training set, feature selection is necessary to avoid a small sample-per-feature ratio and has been shown to provide better classification [24]. The feature selection procedure used in this study consisted of three steps: 1) testing the genes in the training set for significance using the paired *t*-test; 2) re-ranking the top 500 most significant genes/features according to their random-forest importance value - for a given feature, this value reports the standardized drop in prediction accuracy when the class labels are permuted [25], and 3) finding the optimal number of features - by subsequently adding 10% of the features at a time in a top-down forward-wrapper approach starting with the top two features of the ranked list; at each increment the classification accuracy of the training samples was assessed using LOPOCV in a nested loop [26]. Fisher’s exact test was used to calculate the significance of the classification results.

All calculations were performed using the open source R-environment. The R packages randomForest and e1071 were implemented for the random forest importance ranking and SVM-based classification, respectively. Differential survival in the predicted subgroups of samples was demonstrated by Kaplan-Meier plots and tested by the log-rank test. The Cox proportional hazards regression model was used to estimate the hazard ratio (HR), with a 95% CI. The assumption of hazard proportionality for the model was tested. Logistic regression analysis was performed to examine the impact of age at diagnosis, tumor size and grade on the classification predictions and considered significant if the *P*-value was ≤0.05.

We performed molecular subtype classification using the 50-gene classifier described by Parker *et al*. [27]. All 50 genes included in the prediction analysis of microarray (PAM)50 classifier could be mapped to the Agilent platform used. Distances to each of the five subtype centroids were calculated using Spearman’s rank correlation (R package genefu); the nearest centroid classified the subtype of all 164 samples.

### Optimal lncRNA gene signatures

The above-mentioned classification resulted in the same number of different models and lncRNA gene sets as the number of pairs unsuitable for validation in independent datasets. To obtain optimal gene profiles for validation purposes, we used the entire dataset for feature selection as described above. We evaluated the performance of the optimal classifier using LOPOCV by adding one feature at a time in a top-down selection starting with the top two features of the ranked genes, thereby, optimizing the number of genes for obtaining 90% sensitivity, together with the highest specificity with a specified gene list.

Heatmaps of the optimal lncRNA profiles were used to visualize the patterns of expression in the different samples. The normalized log ratios from the lncRNA list were mean-centered within each lncRNA. We produced all heatmaps in Qlucore Omics Explorer 2.3 (Qlucore, Lund, Sweden).

### Validation in independent datasets and comparison to the MammaPrint signature

To examine the performance of the lncRNA profiles, we identified two gene expression datasets measured with Affymetrix HG-U133A + B array containing lncRNAs on a comparable breast cancer patient cohort and therefore suitable for validation [28,29]. We re-annotated the probes as described for the Agilent microarray platform. The Miller dataset consisted of 236 breast cancer patients. Of these, 149 patients were lymph node-negative, 135 of whom had not received systemic adjuvant therapy, and 55 patients died because of breast cancer. The Pawitan dataset consisted of 159 breast cancer patients, most of whom had received systemic adjuvant therapy and 29 died because of breast cancer. The performance of the lncRNA profiles in the independent validation dataset was evaluated using the LOOCV procedure described above. The prognostic potential of the highest ranked lncRNAs of the lncRNA profiles was further investigated in the independent datasets using Kaplan-Meier plots and was tested by the log-rank test. To examine the performance of the MammaPrint signature in our dataset, we performed classification using the 70 genes from the MammaPrint signature, with a threshold providing at least 90% sensitivity, together with the highest specificity.

### Functional implications of individual lncRNAs

In order to associate functional gene sets to each lncRNA in the top of the profiles, we performed gene set enrichment analysis (GSEA). We used the highest ranked lncRNA of the profiles, and computed the Pearson correlation coefficient for each lncRNA-mRNA combination. mRNAs were then ranked according to the Pearson correlation coefficient to generate ranked gene lists for GSEA. GSEA was performed by the JAVA program [30] using the MSigDB C2 CP: REACTOME gene set collection (674 gene sets). Gene sets with a false discovery rate (FDR) value <0.05 after performing 1,000 permutations were considered significant [31].

Relative expression levels of the top lncRNAs, in different subtypes, both in our dataset and in the Affymetrix validation datasets were visualized by box plots and tested for significant associations with the molecular subtypes using the *t*-test. All plots and tests were performed in Qlucore Omics Explorer 2.3 (Qlucore).

## Results

Re-annotation resulted in identification of 4,810 lncRNA probes on the Agilent array. Further analysis was performed using only the 4,810 probes covering 2,811 unique lncRNAs.

### Classification of tumor samples

We performed genome-wide gene expression analysis on frozen tumor biopsies from 164 patients with primary invasive breast cancer. All patients included in the study were adjuvant-naïve, lymph node-negative and had a tumor measuring less than 5 cm; the selected group of non-metastasizing patients had a mean follow up of 18.1 years. Table 1 summarizes the clinical and pathological parameters of patients and their tumors.

Our primary objective was to investigate whether lncRNAs had a prognostic expression pattern that defined the metastatic phenotype. We achieved this goal by developing an SVM classifier; the accuracy of the classifier was the measure of how successful the method was at assigning samples to the correct class. To perform classification, we used 162 samples as training set and performed testing on the remaining matched pair. Repeating this procedure for all matched pairs resulted in cross-validation of all samples and a predicted probability for each patient of having a poor outcome.

**Figure 1:**
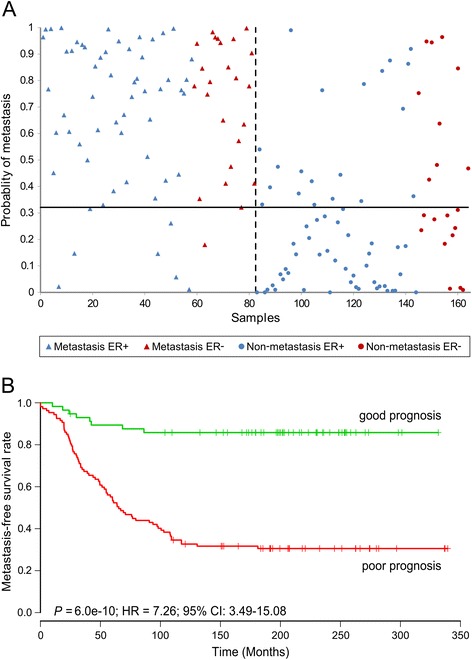
Classification and survival analysis within all samples. **(A)** Dot plot of the overall classification (82 pairs of samples) illustrating the probability of metastasis plotted versus the tumor number (*P* = 7.3e-14). The dashed vertical line separates the patients with metastasis (left of the line) from the non-metastatic patients (right of the line). The horizontal line refers to the discriminating limit; hence, the upper left and lower right corners contain the correctly classified patients. **(B)** Kaplan-Meier survival curve of metastasis-free survival according to model-based prediction using the overall classification. ER, estrogen receptor; HR, hazard ratio.

In Denmark, systemic adjuvant therapy is offered to patients with high risk of recurrence, defined as a cumulative risk of 10% or more of recurrence of breast cancer within 10 years [32]. Therefore, the patient group eligible for adjuvant systemic therapy needs to be classified with a sensitivity ≥ 90%, e.g. misclassification of no more than 8 patients out of the 82 metastatic patients. This sensitivity threshold resulted in an overall classification (82 pairs of samples) that correctly classified 74 out of 82 metastatic samples as having poor prognosis and 53 out of 82 non-metastatic samples as having good prognosis (Figure 1A). Thus, the sensitivity was 90%, specificity 65% and accuracy 77% (Table 2). Furthermore, the Kaplan-Meier analysis for metastasis-free survival (MFS) demonstrated a highly significant difference between the groups predicted to have good or poor prognosis (*P* = 6.0e-10, HR = 7.26, 95% CI 3.49, 15.08) (Figure 1B).

**Table 2:** **Overall classification, estrogen receptor (ER)-positive classification, ER-negative classification and classification using the MammaPrint profile**

**Profile**	**Number of samples (metastasis/non-metastasis)**	**Sensitivity (true positive)**	**Specificity (true negative)**	**Accuracy** ^**a**^	***P*** ^**b**^
**LncRNA classification**					
**Overall classification**	82/82	90 (74)	65 (53)	77	7.3e-14
**ER-positive classification**	55/55	91 (50)	64 (35)	77	1.1e-9
**ER-negative classification**	17/17	94 (16)	0 (0)	47	0.50
**MammaPrint classification**					
**All samples**	82/82	90 (74)	22 (18)	56	0.03
**ER-positive samples**	55/55	91 (50)	55 (30)	73	1.9e-7
**ER-negative samples**	17/17	94 (16)	0 (0)	47	0.50

^a^Mean of sensitivity and specificity. ^b^Fisher’s exact test, one-tailed. Classification performances were assessed by leave one pair out cross-validation with a threshold that resulted in ≥90% sensitivity and maximized the specificity.

The prognostic classifier was developed in a majority of ER-positive samples. Our evaluation of the overall classification performance within the ER-positive and ERnegative samples revealed better specificity of 68% in the ER-positive samples, whereas the specificity in the ER-negative samples was 55% (Additional file 1: Table S1).

Because all the tumor samples included in the study were collected more than a decade ago, no information about human epidermal growth factor (HER)2 status was available in the DBCG database and it was not possible to match samples according to HER2 status. Prediction of intrinsic molecular subtypes revealed unequal distributions of subtypes within the metastatic and non-metastatic patient groups. The luminal A subtype was overrepresented in the non-metastatic group and the luminal B subtype was overrepresented in the metastatic group. The HER2-enriched subtype was also overrepresented in the metastatic group, while the basal-like subtype was underrepresented. In the original study by Parker *et al*., the normal-like class was represented using normal breast tissue [27], indicating that the samples predicted as normal-like most likely contain a large component of normal tissue. Nevertheless, evaluation of the overall classification (82 pairs of samples) performance in the predicted intrinsic molecular subtypes revealed similar overall accuracies in all molecular subtypes (Additional file 1: Table S1).

The overall classification (82 pairs of samples) seemed to be confounded by the influence of ER status, despite matching of the samples. Because of the matched design, it was not possible to perform multivariate analysis to test whether the metastatic outcome in the samples was correlated to any of the traditional clinical variables. Instead, we performed logistic regression analysis to examine whether the prediction accuracies were independent of the traditional clinical variables; age, tumor size, grade, and ER status (Table 3). We found that neither ER status nor any of the other variables influenced the prediction accuracies significantly. Furthermore, we investigated whether the relative expression levels of the top 10 lncRNAs from the overall profile were associated with molecular subtypes. Nine lncRNAs were not associated with molecular subtypes, and only *HOXA11-AS* was significantly associated with the HER2 subtype (Additional file 2: Figure S1).

**Table 3:** **Logistic regression analysis of overall classification and estrogen receptor (ER)-positive classification results on the traditional clinical variable and time to metastasis**

**Overall classification**	**Metastatic patients (n = 82)**		**Non-metastatic patients (n = 82)**	
	**Odds ratio** ^**a**^	***P*** ^**a**^	**Odds ratio** ^**a**^	***P*** ^**a**^
**Age** (≤50 versus >50 years)	1.004	0.97	0.914	0.57
**Tumor size** (range 6 mm to 50 mm)	1.002	0.56	0.990	0.19
**Grade** (range 1 to 3)	1.057	0.33	0.971	0.75
**ER status** (negative versus positive)	1.002	0.98	1.095	0.56
**Time to metastasis** (range 0 to 181 months)	1.001	0.41	-	-
**ER-positive classification**	**Metastatic patients (n = 55)**		**Non-metastatic patients (n = 55)**	
	**Odds ratio** ^**a**^	***P*** ^**a**^	**Odds ratio** ^**a**^	***P*** ^**a**^
**Age** (≤50 versus >50 years)	1.088	0.49	0.526	**0.053**
**Tumor size** (range 6 mm to 50 mm)	0.996	0.34	0.991	0.36
**Grade** (range 1 to 3)	1.084	0.21	1.051	0.65
**Time to metastasis** (range 5 to 131 months)	1.000	0.86	-	-

^a^Logistic regression analysis.

To test whether the predictions would be improved in a more homogeneous sample group, we stratified the samples into 55 ER-positive and 17 ER-negative pairs. We built new classifiers in each group, using the same settings in the SVM algorithm as in the overall classification. We determined the ER status by the *ESR1* expression to obtain the missing values in the DBCG database (Additional file 2: Figure S2). We failed to match ten pairs of samples correctly for ER status (according to *ESR1* expression) and omitted them in the development of new classifiers. Furthermore, this stratification should minimize the influence from the molecular subtypes, especially the effect of the HER2-enriched samples.

**Figure 2:**
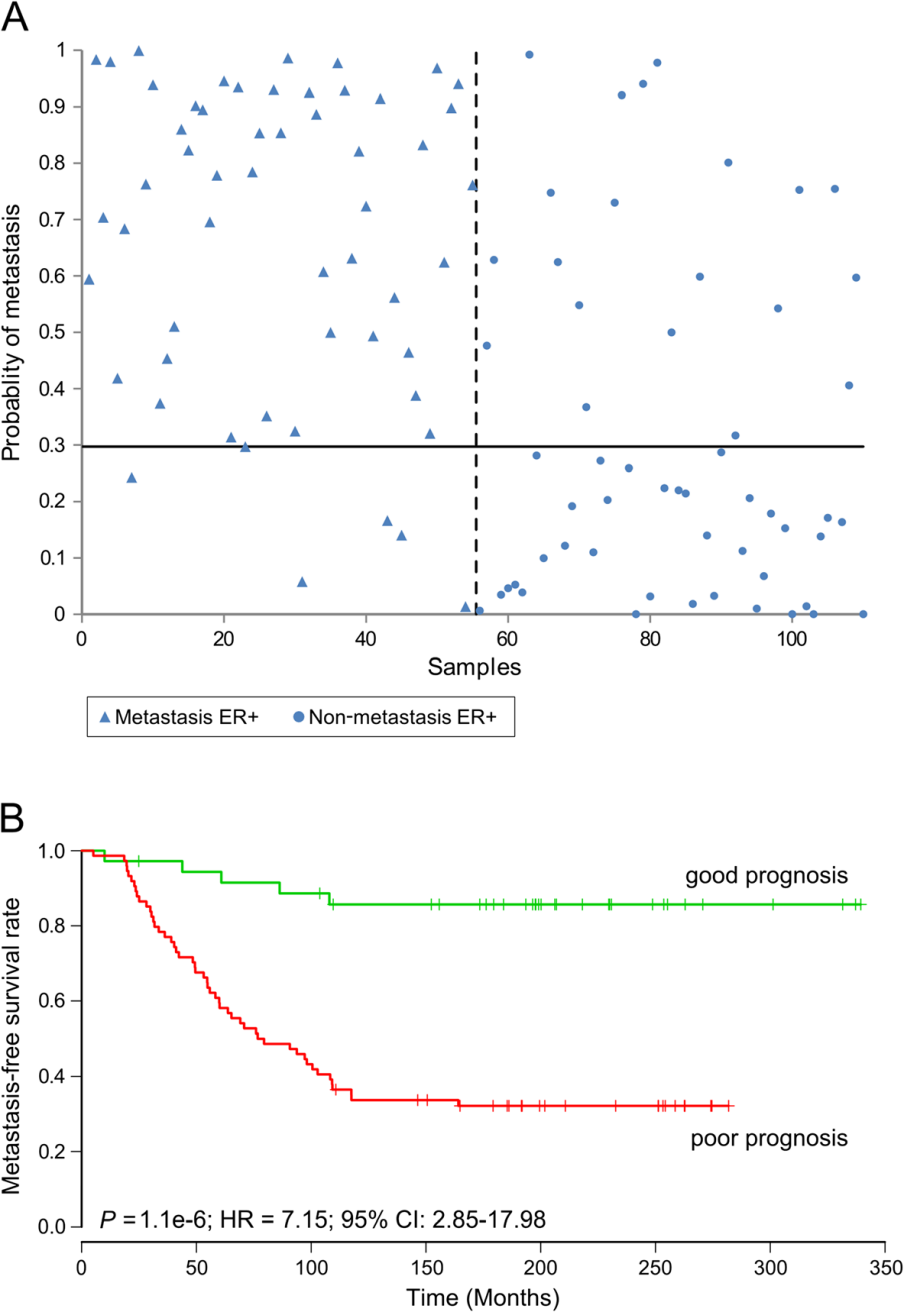
Classification and survival analysis within estrogen receptor (ER)-positive samples. **(A)** Dot plot of the ER-positive classification (55 pairs of samples), illustrating the probability of metastasis plotted versus the tumor number (*P* = 1.1e-9). The dashed vertical line separates the patients with metastasis (left of the line) from the non-metastatic patients (right of the line). The horizontal line refers to the discriminating limit; hence, the upper left and lower right corners contain the correctly classified patients. **(B)** Kaplan-Meier survival curve of metastasis-free survival according to model-based prediction using the ER-positive classification. HR, hazard ratio.

Using the 90% sensitivity threshold, the ER-positive (ER+) classification (55 pairs of samples) correctly classified 50 out of 55 metastatic samples as demonstrating poor prognosis, and 35 out of 55 non-metastatic samples as demonstrating good prognosis, hence, demonstrating a sensitivity of 91% and a specificity of 64% (accuracy 77%) (Figure 2A, Table 2). In addition, the Kaplan-Meier analysis for MFS demonstrated a highly significant difference between the groups predicted to have good or poor prognosis (*P* = 1.1e-6, HR = 7.15, 95% CI 2.85, 17.98) (Figure 2B).

The ER-negative (ER-) classification (17 pairs of samples), correctly classified 16 out of 17 metastatic samples as having poor prognosis, and 0 out of 17 non-metastatic samples as having good prognosis. Thus, the accuracy was 47% and insignificant (Table 2).

Once more, we performed logistic regression analysis to examine whether the prediction accuracies in the ER-positive classification were independent of the traditional clinical variables; age, tumor size, and grade (Table 3). The test revealed a trend towards significant dependency of age in the non-metastatic patients (*P* = 0.053, odds ratio (OR) = 0.526). We also examined whether a shorter or longer time to metastasis (range 5 to 131 months) influenced the prediction accuracies of the classifier and found that the classifications were independent of time to metastasis.

Evaluations of the ER-positive and ER-negative classifications within the intrinsic molecular subtypes (Additional file 1: Table S1) showed a very small difference in performance between the luminal subtypes in the ER-positive classification, although only two or three samples represented the difference, due to the small sample size.

Classification with the 70 genes from the MammaPrint profile, using the 90% sensitivity threshold in all our samples, resulted in a specificity of 22%. Classification of the ER-positive samples provided sensitivity of 91% and specificity of 55%, whereas the MammaPrint profile failed to classify any non-metastasizing ER-negative samples (Table 2).

### Optimal lncRNA profiles

Because the classifications provided slightly different lncRNA sets in each round of cross-validation, we generated an optimal lncRNA set by building new models in all pairs (Additional file 2: Figure S3A) and in all ER-positive pairs (Additional file 2: Figure S3B), and LOPOCV was used to determine the optimal length of the lncRNA profiles.

The resulting optimal lncRNA profiles consisted of 47 and 168 lncRNA probes in all pairs and in ER-positive pairs of samples, respectively (Additional file 1: Tables S2 and S3, Additional file 2: Figures S4 and S5). The overall and the ER-positive profiles had 31 overlapping lncRNA probes (Additional file 1: Tables S2 and S3).

### Identification of associated biological pathways

GSEA pathway analysis using mRNA ranked according to lncRNA correlation revealed a significant enriched pathway associated with two of the top lncRNAs (FDR < 0.01) (Additional file 1: Table S5). The nuclear enriched abundant transcript 1 (*NEAT1*) was associated with several pathways involved in RNA polymerase I promoter opening and transcription. The *HOXA11* antisense RNA (*HOXA11-AS*) was associated with collagen formation pathways and extracellular matrix organization pathways.

### External validation

**Figure 3:**
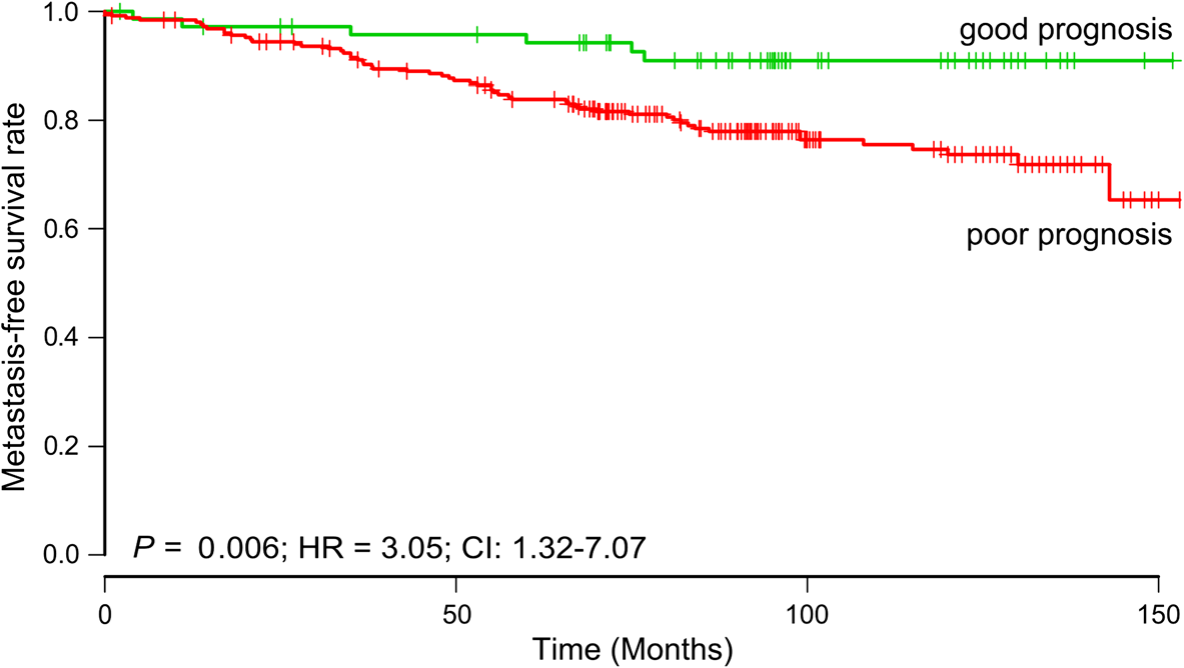
Survival analysis of the estrogen receptor (ER)-positive profile in independent samples. Kaplan-Meier curve of metastasis-free survival according to model-based prediction using the Affymetrix probesets (n = 59) covering the ER-positive profile. ER-positive breast cancer patients from two different datasets were analyzed (n = 324). HR, hazard ratio.

To pursue an external validation of our lncRNA profiles in independent datasets, we examined two existing Affymetrix datasets [28,29]. We focused on re-annotating the Affymetrix U133A + B array because of its higher abundance of probes covering the entire transcriptome, including a selection of lncRNAs. Unfortunately, only 15 lncRNAs from the overall 47-gene profile were covered by 20 probesets on the Affymetrix U133A + B array. Fifty-nine lncRNAs from the ER-positive 168-gene profile were covered by 75 probesets on the Affymetrix U133A + B array, which was presumed to reduce power of the external validation. We found that the ER-positive profile could be validated in the ER-positive samples in the Miller dataset combined with the Pawitan dataset (324 samples) with an accuracy of 58%. This result was significant (*P* = 0.003) (Additional file 1: Table S4), despite the fact that the Affymetrix dataset only covered 59 of the lncRNAs in the overall 168-gene profile (Additional file 1: Table S3). Furthermore, survival analysis of the ER-positive profile in the independent samples showed that it could separate patients with respect to probability of MFS (*P* = 0.006; HR = 3.05; 95% CI 1.32, 7.07) (Figure 3).

In the independent samples, we also investigated the prognostic potential of some of the individual lncRNAs, found in the top of our profiles. Kaplan-Meier survival analysis showed that expression levels of four out of seven of the investigated lncRNAs significantly correlated with unfavorable MFS in the validation samples (*P* <0.05) (Additional file 2: Figure S6).

## Discussion

The aim of the study was to identify breast cancer patients who are overtreated using the current protocol. We have analyzed lncRNA expression in 164 primary breast tumors by microarray gene expression analysis and showed for the first time that a profile consisting exclusively of lncRNAs is associated with the risk of metastases in lymph node-negative breast cancer patients. The patients included in our study had not received any adjuvant treatment because most of them were diagnosed 20 to 30 years ago. By using gene expression data and an SVM algorithm, we were able to generate lncRNA classifiers that predicted metastasis outcome with high accuracy.

Typically, retrospective studies are often biased towards patients with available tumor material, which again is related to tumor size and outcome [33]. We employed a matched design that increased the power of the predictor by enriching for informative clinical endpoints compared to a cohort study; furthermore, we avoided classifications that were dependent on the traditional clinical variables. Matching the samples also minimized bias related to storage time, sampling method, and diagnostic procedures. We reduced the technical variation during the purification and microarray procedure by processing the matched pair concurrently.

Many studies have developed microarray-based gene expression profiles consisting primarily of mRNA from frozen tumors from patients with good or poor prognoses and predicted the metastasis risk by overall gene expression analysis of the primary tumor [1-4,34]. Many of these signatures are not purely prognostic, because they were developed in a mixture of patients who were treated with or without systemic therapy. Additionally, validation studies have demonstrated that the prognostic accuracy is strongly time-dependent in several of these signatures and their use might be better suited to predict early relapse [35,36]. In the present data, the very long follow-up time resulted in very reliable information about outcomes in our patients and provided strength to the classifier for predicting both early and late metastasis events. The logistic regression analysis showed that the classifiers predictions were independent of time to event.

To explore whether lncRNAs could be used to predict metastatic outcomes in early breast cancer, we re-annotated our Agilent platform and found 4,810 probes covering 2,811 unique lncRNAs. This Agilent array is particularly enriched for lncRNAs and useful for studying the lncRNA transcriptome. Using only the lncRNA gene expression data, we performed the LOPOCV method to estimate the prediction performance in our samples. Both the overall classification and the ER-positive classification resulted in an accuracy of 77%.

However, we observed differences in prediction performance in ER-positive and ER-negative subgroups. The classification performance was low in ER-negative samples in the non-metastasizing group of patients, which was expected because the model was developed in a majority of ER-positive samples. In general, most prognostic profiles developed in heterogeneous patient cohorts contain proliferation-related genes and possess prognostic power only in ER-positive and HER2-negative tumors, because proliferation is the major determinant of prognosis in this subgroup of patients [37,38].

To test whether classification in ER-positive samples alone would improve the specificity, we stratified the pair of samples according to ER status, determined by the *ESR1* expression. Microarray-based determination of ER status is a reliable measure to predict immunohistochemistry-based ER status [39]. Dividing the dataset further into intrinsic molecular subtypes would have resulted in very small datasets.

The classification specificity within the ER-positive samples was comparable to the overall classification; the prediction gene lists developed in both classifications had many lncRNAs in common. It further demonstrated the high magnitude of the majority of samples during classifier development. Thus the ER-negative samples did not affect the overall classification within the ER-positive samples.

Most interestingly, we found that the classifications were independent of most of the traditional prognostic markers, including ER status, tumor size, and grade, revealing that the lncRNAs provide additional prognostic information beyond the classical parameters. Furthermore, we found that short or long time to event had no influence on the predictions, which is of major importance, especially in ER-positive cancer, where the risk of death from the tumors persists for 20 years [40]. ER positivity is only associated with a more favorable prognosis during the first 5 years after diagnosis; studies with longer follow-up time have shown equal survival rates among ER-positive and ER-negative patients [41].

In addition, the classifications appeared to be subtype-independent, although the distribution of luminal A and luminal B subtypes was dissimilar, with a predominance of the luminal B subtype in the metastatic patients and a predominance of the luminal A subtype in the non-metastatic patients.

The classification accuracy within the ER-negative samples was not significant; this could be due to the small number of samples analyzed and the interference of molecular subtypes. Other studies have shown the difficulties in identification of good prognosis ER-negative cancer patients, especially triple-negative basal-like cancers [42]. Conversely, a larger study using 186 untreated ER-negative basal-like patients found that high expression of immune response genes gave a better outcome than ER-negative cancers with low expression [43].

Examination of associated functional pathways revealed that lncRNAs included in the profile are involved in RNA polymerase I transcription and extracellular matrix organization. Several studies have shown that genes involved in extracellular matrix organization and collagen formation are important players, when cancers cells metastasize to distant sites and, therefore, are closely related to the survival time of breast cancer patients [44].

The pathways associated with *NEAT1* included purely histone cluster genes indicating that *NEAT1* could be involved in epigenetic modifications. In prostate cancer *NEAT1* are involved in gene transcription of cancer progression genes by interacting with histones and/or chromatin-modifying proteins [45], which supports our findings.

Validation of the identified lncRNA profiles in independent datasets was challenged by the lack of suitable lncRNA datasets. Even though only 59 out of 168 lncRNAs were present in the independent validation dataset, we sought to validate our ER-positive profile. Despite the incomplete coverage of lncRNAs and the fact that many of these patients were lymph node-positive and had been treated with adjuvant therapy, we were able to obtain significant prediction of metastasis. Furthermore, the classification results were significantly associated with MFS. These results indicate a true prognostic value of lncRNA expression in breast cancer.

We performed survival analysis to further investigate the prognostic potential of some of the lncRNAs individually in the validation datasets. We found that high expression of *NEAT1* in the ER-negative validation samples correlated with poor survival. A similar association has also been demonstrated in a large cohort of both ER-positive and ER-negative breast cancer patients [46]. High expression of TOPORS antisense RNA 1 (*TOPORS-AS1*) was associated with good outcome in the ER-positive validation samples and with poor outcome in the ER-negative validation samples. *TOPORS-AS1* has previously been associated with good outcome in breast cancer patients and the authors suggested that *TOPORS-AS1* acts as a tumor suppressor [47]. *TOPORS-AS1* could potentially work as a prognostic biomarker, although the association with hormone receptor status needs to be further investigated. The lncRNA *RP11-539 L10.3* was also associated with good outcome in the ER-positive validation samples. This lncRNA was the most significant prognostic marker in our ER-positive samples; however, additional studies are needed to clarify the significance of *RP11-539 L10.3*. High *HOX11-AS* was associated with poor outcome in the ER-positive validation samples, an association not previously reported. *HOXA11-AS* has been proposed to negatively regulate *HOXA11* mRNA levels in the human endometrium [48].

Previous studies have conducted data mining of Affymetrix arrays for lncRNAs [49-51]. We re-annotated the probes at the Affymetrix 133A + B array to identify the probesets covering the lncRNAs in the classifiers. We identified two different datasets from these Affymetrix chips containing breast cancer samples and validated the profiles in different subgroups of samples from these two datasets. However, only about one third of the lncRNA probes in the profiles could be mapped to probesets at the Affymetrix array, impeding the validation.

Several other sources of variation affect the outcome when transferring gene sets from one dataset to another. Differences in RNA extraction, amplification, labeling, size of oligonucleotides, sequence variation and hybridization procedures, as well as differences in data pre-processing and biological variation within patient samples represent major challenges [52]. Previous studies have shown the difficulties of external validation in datasets from different microarray platforms and the performances of the classifiers dropped dramatically [53,54].This study is the first to assess whether lncRNAs can be used for prognostic profiling in breast cancer. Several other studies have investigated gene expression data or RNA sequencing data and identified single lncRNAs with prognostic power or cancer progression properties [16,19]. Du *et al*. re-annotated Affymetrix arrays and identified relevant lncRNAs that were associated with cancer subtypes and clinical prognosis in prostate cancer, glioblastoma, ovarian cancer and lung squamous cell carcinoma [50]. Another study found significant RNA profiles, comprising both mRNAs and lncRNAs, which were correlated with primary and metastatic ductal pancreatic adenocarcinoma [55].

The clinical relevance of the current study is to support a more accurate prognosis and thereby reduce the use of adjuvant therapy in lymph node-negative breast cancer patients. The challenge is to obtain a higher specificity than the traditional markers currently used. A retrospective study has shown that the treatment guidelines provided by the St Gallen consensus criteria [56] and the online tool, Adjuvant! Online (standard version 8.0), assigned very few patients to the low-risk group, providing sensitivity above 90% at the expense of specificity below 20% [57]. We obtained slightly higher specificity in our study (65% and 64%), when comparing our classification results with the prognostic performances of different prognostic gene profiles. The Dutch MammaPrint profile initially showed specificity of 59% [2], however this dropped to 42% in a validation study [58]. The MammaPrint profile performed worse than expected in our dataset, probably due to the large number of ER-negative non-metastasizing patients. The performance of MammaPrint improved when stratifying patients by ER status, demonstrating highly significant performance in ER-positive samples, whereas, the prognostic significance of the MammaPrint profile in ER-negative patients is questionable. Notably, our ER-positive lncRNA profile improved the specificity by 9% compared to the MammaPrint profile. Our profiles, if further confirmed, could therefore result in a substantial reduction of the number of lymph node-negative patients who are recommended to have unnecessary systemic adjuvant therapy.

## Conclusions

We used primary tumors to develop prognostic profiles consisting of lncRNAs that predict metastasis in lymph node-negative breast cancer patients independently of the traditional clinical markers such as tumor size, grade, and ER status. To our knowledge, this is the first study that demonstrates that lncRNA profiles can distinguish metastatic patients from non-metastatic patients with sensitivity above 90% and specificity of 64 to 65%. The patients included in the study had not received any kind of adjuvant treatment; hence the performance of the profiles was not influenced by treatment response. Further analysis within ER-negative and ER-positive samples revealed similar prediction accuracy in ER-positive breast cancer samples, whereas we did not have statistical power to assess a potential prognostic value of lncRNAs in ER-negative cancers.

We managed to validate the prognostic value of our ER-positive profile in two independent breast cancer datasets, although these datasets were created with a completely different type of microarray. Further validation in a dataset from the same type of microarray platform or validation using PCR-based methods or target RNA sequencing is necessary.

## Additional files

Additional file 1: Table S1. Evaluation of the overall classification, the estrogen receptor-(ER)-positive and ER-negative classification within molecular subtype sample subsets. Table S2 The overall 47-gene profile, corresponding to 45 unique long non-coding RNAs (lncRNAs). The colored GENCODE IDs depict the 31 lncRNAs that overlap with the ER-positive profile. Table S3 The ER-positive 168-gene profile, corresponding to 140 unique lncRNAs. The colored GENCODE IDs depict the 31 lncRNAs that overlap with the overall profile. Table S4 External validation in independent ER-positive samples (n = 324) using the ER-positive profile. Table S5 Gene set enrichment analysis (GSEA) identifies highly significant pathways associated with top lncRNAs from the overall profile. Additional file 2: Figure S1. Box plots showing **(A)** the relative expression levels of the individual long non-coding RNAs (lncRNAs) from the top-10 list of the overall classification in the different subtypes and **(B)** the relative expression levels of the individual lncRNAs in the independent data sets in the different subtypes. Figure S2 ESR1 gene expression measurements were used to determine estrogen receptor (ER) status. Figure S3 **(A)** Optimization of the overall classification, resulting in an optimized number of lncRNAs to include in the overall profile (the simplest optimal profile consisted of 47 genes) and **(B)** optimization of the ER-positive classification, resulting in an optimized number of lncRNAs to include in the ER-positive profile (the simplest optimal profile consisted of 168 genes). We based the mean balanced accuracy on 100 support vector machine (SVM) models in each data point. Figure S4 Expression data matrix of the overall 47-gene profile visualized as a heatmap. Each column represents a tumor sample and each row an lncRNA. Tumor samples are ordered according to their classification probability estimate from 0 to 1. The 47 lncRNAs are order via hierarchical clustering. The metastatic status and ER status are shown in the top bar. Figure S5 Expression data matrix of the ER-positive 168-gene profile visualized as a heatmap. Each column represents a tumor sample and each row an lncRNA. Tumor samples are ordered according to their classification probability estimate from 0 to 1. The 168 lncRNAs are ordered via hierarchical clustering. The metastatic status is shown in the top bar. Figure S6 Kaplan-Meier survival curves of MFS for all validation samples (n = 395) and divided into ER-positive samples (n = 324) and ER-negative samples (n = 71).

## Abbreviations

DBCG: Danish Breast Cancer Cooperative Group
ER: estrogen receptor
GSEA: gene set enrichment analysis
HER: human epidermal growth factor
HR: hazard ratio
lncRNA: long non-coding RNA
LOPOCV: leave one pair out cross-validation
MFS: metastasis-free survival
SVM: support vector machine
