# Supplementary material for: Long non-coding RNA expression profiles predict metastasis in lymph node-negative breast cancer independently of traditional prognostic markers
Source: Breast Cancer Res. 2015 Apr 11;17(1):55. doi: 10.1186/s13058-015-0557-4 (PMC4416310; doi:10.1186/s13058-015-0557-4)
Supplement: Additional file 1: Table S1. — Evaluation of the overall classification, the estrogen receptor-(ER)-positive and ER-negative classification within molecular subtype sample subsets. Table S2 The overall 47-gene profile, corresponding to 45 unique long non-coding RNAs (lncRNAs). The colored GENCODE IDs depict the 31 lncRNAs that overlap with the ER-positive profile. Table S3 The ER-positive 168-gene profile, corresponding to 140 unique lncRNAs. The colored GENCODE IDs depict the 31 lncRNAs that overlap with the overall profile. Table S4 External validation in independent ER-positive samples (n = 324) using the ER-positive profile. Table S5 Gene set enrichment analysis (GSEA) identifies highly significant pathways associated with top lncRNAs from the overall profile. [file 13058_2015_557_MOESM1_ESM.pdf]

Supplementary Tables

Table S1. Evaluation of the overall classification, the ER+ and ER- classification within molecular subtype sample subsets

	No. of samples (Metastasis/Non-metastasis)	Sensitivity (TP)	Specificity (TN)	Accuracy [a]	P [b]
**Overall classifier**	82/82	90 (74)	65 (53)	77	7.3e-14
ER+	58/62	88 (51)	68 (42)	78	2.3e-10
ER-	24/20	96 (23)	55 (11)	75	2.0e-4
Basal-like	11/16	91 (10)	56 (9)	74	1.6e-2
HER2-enriched	14/6	100 (14)	50 (3)	75	1.8e-2
LumA	20/36	95 (19)	69 (25)	82	1.8e-6
LumB	36/21	83 (30)	71 (15)	77	5.3e-5
Normal-like	1/3	100 (1)	33 (1)	67	0.75
**ER+ classifier**	55/55	91 (50)	64 (35)	77	1.1e-9
Basal-like	1/0	100 (1)	-	-	
HER2-enriched	2/0	100 (2)	-	-	
LumA	20/35	90 (18)	69 (24)	79	2.3e-5
LumB	32/18	91 (29)	56 (10)	73	6.6e-4
Normal-like	0/2	-	50 (1)	-	
**ER- classifier**	17/17	94 (16)	0 (0)	47	0.50
Basal-like	7/13	86 (6)	0 (0)	43	0.35
HER2-enriched	8/4	100 (8)	0 (0)	50	1
LumB	1/0	100 (1)	-	-	
Normal-like	1/0	100 (1)	-	-	

[a] Mean balanced accuracy
[b] Fisher's exact test, one-tailed
Abbreviations: TP, true positive; TN, true negative

Table S2. Overall 47-gene profile, corresponding to 45 unique lncRNAs; colored GENCODE IDs depict the 31 lncRNAs that overlap with the ER positive profile

GENCODE ID	Gene biotype	Ensembl gene name	Affymetrix U133B probe ID
ENSG00000250132.1	processed_transcript	RP11-359B12.1	236042_at
ENSG00000177738.3	processed_transcript	CTD-2201E18.3	
ENSG00000233016.1	antisense	SNHG7	229050_s_at
ENSG00000245532.3	lincRNA	NEAT1	225239_at
ENSG00000235453.3	processed_transcript	TOPORS-AS1	221979_at, 78383_at
ENSG00000251580.1	antisense	RP11-539L10.3	229189_s_at, 229190_at
ENSG00000240990.5	antisense	HOXA11-AS	230666_at
ENSG00000243243.1, ENSG00000237813.3	antisense	AC073130.3, AC002066.1	
ENSG00000253582.1	antisense	RP11-649G15.2	
ENSG00000175772.10	processed_transcript	AC112229.7	235083_at
ENSG00000224164.1	lincRNA	RP3-369A17.4	
ENSG00000232790.2	processed_transcript	AC006481.1	
ENSG00000260025.1	lincRNA	RP11-490M8.1	236656_s_at, 236657_at
ENSG00000235740.1	antisense	RP11-436I24.1	
ENSG00000235385.1	lincRNA	GS1-600G8.5	
ENSG00000231683.2	processed_transcript	RP1-27K12.2	
ENSG00000260630.2	antisense	SNAI3-AS1	
ENSG00000235142.2	processed_transcript	RP1-60O19.1	230432_at, 235774_at
ENSG00000260071.1	lincRNA	RP11-418I22.2	
ENSG00000243944.1	processed_transcript	RP11-167H9.4	
ENSG00000224271.1	processed_transcript	RP11-191L9.4	
ENSG00000267313.2	lincRNA	RP11-142I20.1	
ENSG00000196364.7	lincRNA	RP11-616M22.6	
ENSG00000229637.2	antisense	HOXB-AS5	230845_at
ENSG00000034063.9	processed_transcript	UHRF1	225655_at, 207730_x_at
ENSG00000263753.1	lincRNA	LINC00667	
ENSG00000260231.1	antisense	RP4-659J6.2	232579_at
ENSG00000251095.2	antisense	RP11-115D19.1	
ENSG00000223745.2	processed_transcript	RP4-717I23.3	
ENSG00000233006.2	processed_transcript	AC034220.3	
ENSG00000232284.3	lincRNA	GNG12-AS1	
ENSG00000226906.1	lincRNA	TTTY4	234913_at
ENSG00000236008.1	lincRNA	AC011747.4	
ENSG00000260852.1	antisense	FBXL19-AS1	
ENSG00000258441.1	processed_transcript	LINC00641	
ENSG00000179818.7	antisense	PCBP1-AS1	
ENSG00000214719.5	processed_transcript	AC005562.1	
ENSG00000224655.1	lincRNA	AC018737.3	
ENSG00000268240.1	lincRNA	RP11-678G14.4	
ENSG00000224078.5	processed_transcript	SNHG14	241834_at
ENSG00000223745.2	processed_transcript	RP4-717I23.3	
ENSG00000172965.7	processed_transcript	AC068491.1	
ENSG00000253322.1	lincRNA	RP11-388G22.1	
ENSG00000172965.7	processed_transcript	AC068491.1	
ENSG00000234936.1	antisense	AC010883.5	
ENSG00000259803.1	antisense	SLC22A31	232765_x_at
ENSG00000260192.1	lincRNA	RP11-756H20.1	

Table S3. ER positive 168-gene profile, corresponding to 140 unique lncRNAs; colored GENCODE IDs depict the 31 lncRNAs that overlap with the overall profile

GENCODE ID	Gene biotype	Ensembl gene name	Affymetrix U133B probe ID
ENSG00000251580.1	antisense	RP11-539L10.3	229189_s_at, 229190_at
ENSG00000235453.3	processed_transcript	TOPORS-AS1	221979_at, 78383_at
ENSG00000250132.1	processed_transcript	RP11-359B12.1	236042_at
ENSG00000175772.10	processed_transcript	AC112229.7	235083_at
ENSG00000235142.2	processed_transcript	RP1-60O19.1	230432_at, 235774_at
ENSG00000177738.3	processed_transcript	CTD-2201E18.3	
ENSG00000233016.1	antisense	SNHG7	229050_s_at
ENSG00000224905.2	processed_transcript	AP001347.6	
ENSG00000224271.1	processed_transcript	RP11-191L9.4	
ENSG00000253582.1	antisense	RP11-649G15.2	
ENSG00000232790.2	processed_transcript	AC006481.1	
ENSG00000225470.2	lincRNA	JPX	229315_at
ENSG00000250135.1	processed_transcript	RP4-622L5.2	226976_at
ENSG00000247271.2	processed_transcript	CTD-2003C8.1	
ENSG00000157306.10	processed_transcript	RP11-66N24.4	218540_at
ENSG00000224078.5	processed_transcript	SNHG14	
ENSG00000244649.1	lincRNA	CTD-2377D24.6	
ENSG00000224078.5	processed_transcript	SNHG14	221974_at
ENSG00000196364.7	lincRNA	RP11-616M22.6	
ENSG00000034063.9	processed_transcript	UHRF1	225655_at, 207730_x_at
ENSG00000179818.7	antisense	PCBP1-AS1	
ENSG00000225177.1	antisense	RP11-390P2.4	
ENSG00000254615.2	lincRNA	RP11-395G23.3	235205_at
ENSG00000213963.2	processed_transcript	AC074286.1	231540_at
ENSG00000224164.1	lincRNA	RP3-369A17.4	
ENSG00000249955.1	antisense	RP11-6E9.4	
ENSG00000215256.3	processed_transcript	DHRS4-AS1	227446_s_at
ENSG00000229637.2	antisense	HOXB-AS5	230845_at
ENSG00000263753.1	lincRNA	LINC00667	
ENSG00000188206.5	processed_transcript	HNRNPU-AS1	
ENSG00000214870.4	processed_transcript	AC004540.5	240194_at
ENSG00000172965.7	processed_transcript	AC068491.1	
ENSG00000224078.5	processed_transcript	SNHG14	241834_at
ENSG00000226906.1	lincRNA	TTTY4	234913_at
ENSG00000240990.5	antisense	HOXA11-AS	230666_at
ENSG00000223745.2	processed_transcript	RP4-717I23.3	
ENSG00000267313.2	lincRNA	RP11-142I20.1	
ENSG00000258808.1	processed_transcript	RP11-255G12.3	
ENSG00000247934.3	antisense	RP11-967K21.1	
ENSG00000227036.2	processed_transcript	LINC00511	230812_at, 230858_at
ENSG00000196421.3	lincRNA	LINC00176	
ENSG00000243944.1	processed_transcript	RP11-167H9.4	
ENSG00000253496.2	lincRNA	RP11-13N12.1	
ENSG00000260669.2	processed_transcript	AL136419.6	218571_s_at, 218572_at
ENSG00000255080.1	lincRNA	RP11-1082L8.3	
ENSG00000259084.1	sense_overlapping	RP11-1070N10.6	206413_s_at
ENSG00000225177.1	antisense	RP11-390P2.4	
ENSG00000255267.2	processed_transcript	RP11-430H10.2	
ENSG00000268823.1	lincRNA	CTC-457E21.6	
ENSG00000214851.4	lincRNA	LINC00612	
ENSG00000172965.7	processed_transcript	AC068491.1	
ENSG00000162290.11	processed_transcript	DCP1A	225443_at
ENSG00000254154.3	processed_transcript	RP4-798P15.3	
ENSG00000232685.3	antisense	LINC00442	
ENSG00000226800.5	antisense	CACTIN-AS1	
ENSG00000268230.1	processed_transcript	CTD-2619J13.8	229819_at
ENSG00000245532.3	lincRNA	NEAT1	225239_at

**Table S3 continued**			
ENSG00000206417.4	antisense	H1FX-AS1	
ENSG00000236861.2	antisense	AC006378.2	
ENSG00000232284.3	lincRNA	GNG12-AS1	
ENSG00000255471.1	antisense	RP11-736K20.5	239220_at
ENSG00000267080.1	antisense	ASB16-AS1	
ENSG00000235079.1	antisense	ZRANB2-AS1	
ENSG00000232995.2	processed_transcript	RP11-267N12.3	
ENSG00000235142.2	processed_transcript	RP1-60O19.1	230432_at
ENSG00000232593.1	processed_transcript	RP11-258C19.5	
ENSG00000235703.1	processed_transcript	RP13-507I23.1	232241_at
ENSG00000246130.1	processed_transcript	RP11-875O11.2	234141_s_at
ENSG00000230513.1	antisense	THAP7-AS1	
ENSG00000034063.9	processed_transcript	UHRF1	225655_at, 207730_x_at
ENSG00000204792.2	lincRNA	AC104135.3	
ENSG00000231312.2	processed_transcript	AC007246.3	
ENSG00000262879.1	processed_transcript	RP11-156P1.3	
ENSG00000262086.1	sense_overlapping	RP11-510M2.10	213934_s_at
ENSG00000247092.2	antisense	SNHG10	238691_at, 244786_at
ENSG00000247092.2	antisense	SNHG10	238691_at, 244786_at
ENSG00000250608.1	processed_transcript	RP11-933H2.4	
ENSG00000253656.1	lincRNA	KB-1568E2.1	
ENSG00000246985.2	processed_transcript	SOCS2-AS1	
ENSG00000241181.1	processed_transcript	AC093734.13	235817_at
ENSG00000204362.5	lincRNA	RP11-380J14.1	
ENSG00000260886.1	antisense	RP11-432I5.1	
ENSG00000223745.2	processed_transcript	RP4-717I23.3	244383_at
ENSG00000251095.2	antisense	RP11-115D19.1	
ENSG00000232808.1	antisense	TTTY20	
ENSG00000261824.2	lincRNA	LINC00662	
ENSG00000223745.2	processed_transcript	RP4-717I23.3	
ENSG00000259953.1	sense_overlapping	RP11-4O1.2	
ENSG00000230729.1	antisense	RP11-296L22.8	
ENSG00000260455.1	lincRNA	RP1-67M12.2	241670_x_at
ENSG00000227403.1	processed_transcript	AC009299.3	236451_at
ENSG00000204092.2	lincRNA	RP11-552E20.3	
ENSG00000245532.3	lincRNA	NEAT1	
ENSG00000260455.1	lincRNA	RP1-67M12.2	
ENSG00000223358.1	antisense	EHHADH-AS1	
ENSG00000267279.1, ENSG00000267175.1	lincRNA	RP11-879F14.2 RP11-879F14.1	230574_at
ENSG00000248508.2	processed_transcript	RP11-521C20.4	
ENSG00000259803.1	antisense	SLC22A31	232765_x_at
ENSG00000205890.3	antisense	RP11-473M20.5	
ENSG00000235724.2	antisense	AC009299.2	
ENSG00000205959.3	processed_transcript	RP11-689P11.2	
ENSG00000251532.1	lincRNA	CTD-2245E15.3	
ENSG00000230074.1	antisense	RP11-195F19.9	
ENSG00000230513.1	antisense	THAP7-AS1	236403_at
ENSG00000248980.1	antisense	RP11-87F15.2	
ENSG00000260025.1	lincRNA	RP11-490M8.1	236656_s_at, 236657_at
ENSG00000215403.1	antisense	LL22NC01-81G9.3	
ENSG00000224078.5	processed_transcript	SNHG14	
ENSG00000168367.5	lincRNA	RP11-158I3.2	234925_at
ENSG00000236780.1	lincRNA	AC078941.1	
ENSG00000172965.7	processed_transcript	AC068491.1	
ENSG00000225206.3	lincRNA	MIR137HG	
ENSG00000160695.9	processed_transcript	VPS11	203292_s_at
ENSG00000257151.1, ENSG00000224078.5	lincRNA, processed transcript	RP11-701H24.2 SNHG14	226587_at, 226591_at

Table S3 continued			
ENSG00000262086.1	sense_overlapping	RP11-510M2.10	213934_s_at
ENSG00000258441.1	processed_transcript	LINC00641	
ENSG00000231721.2	processed_transcript	AC058791.2	
ENSG00000215022.2	processed_transcript	RP1-257A7.4	
ENSG00000189295.8	lincRNA	ANKRD62P1-PARP4P3	
ENSG00000250421.1, ENSG00000249894.1	lincRNA	RP11-83M16.6 RP11-434D9.2	
ENSG00000224078.5	processed_transcript	SNHG14	
ENSG00000225470.2	lincRNA	JPX	
ENSG00000224078.5	processed_transcript	SNHG14	
ENSG00000226363.2	lincRNA	AC009336.24	
ENSG00000213981.3	processed_transcript	AC007277.3	
ENSG00000224078.5	processed_transcript	SNHG14	
ENSG00000250802.2	processed_transcript	ZBED3-AS1	
ENSG00000260172.1	lincRNA	RP11-358M11.3	
ENSG00000248508.2	processed_transcript	RP11-521C20.4	
ENSG00000254530.1	lincRNA	RP11-460B17.3	
ENSG00000250903.2	processed_transcript	RP1-80B9.2	
ENSG00000232084.1	lincRNA	AC104782.3	
ENSG00000224078.5	processed_transcript	SNHG14	228370_at
ENSG00000260071.1	lincRNA	RP11-418I22.2	
ENSG00000214870.4	processed_transcript	AC004540.5	240194_at
ENSG00000237187.3	processed_transcript	RP11-65F13.2	229014_at
ENSG00000229989.3	lincRNA	RP11-31E23.1	
ENSG00000267712.1, ENSG00000206129.3	lincRNA	RP11-456O19.4 CTD-2008L17.2	
ENSG00000259560.1	lincRNA	RP11-648K4.2	
ENSG00000267575.1	processed_transcript	CTC-459F4.3	236503_at
ENSG00000258077.1	processed_transcript	RP11-114H23.1	
ENSG00000228784.1	processed_transcript	AC013400.2	
ENSG00000225470.2	lincRNA	JPX	
ENSG00000224078.5	processed_transcript	SNHG14	
ENSG00000228705.1	antisense	LINC00659	
ENSG00000230812.1	lincRNA	RP4-794H19.4	
ENSG00000230163.1	antisense	SMIM12-AS1	
ENSG00000239498.1	lincRNA	AC114765.1	
ENSG00000175611.7	processed_transcript	LINC00476	227893_at
ENSG00000263680.1	processed_transcript	RP11-57A1.1	
ENSG00000259673.1	lincRNA	IQCH-AS1	
ENSG00000234423.1	lincRNA	AC019118.2	
ENSG00000223745.2	processed_transcript	RP4-717I23.3	
ENSG00000234690.2	processed_transcript	AC073283.4	
ENSG00000259091.1	processed_transcript	LINC00517	
ENSG00000260280.1	sense_overlapping	SLX1B-SULT1A4	218317_x_at, 233334_x_at, 222094_at, 222145_at, 214712_at
ENSG00000229852.2	processed_transcript	RP11-398K22.12	
ENSG00000224078.5	processed_transcript	SNHG14	
ENSG00000231881.1	lincRNA	RP5-1120P11.3	234345_at
ENSG00000251095.2	antisense	RP11-115D19.1	233413_at
ENSG00000256392.1, ENSG00000212694.4	processed_transcript, lincRNA	RP11-347I19.3 AC084018.1	226369_at, 210679_x_at
ENSG00000224597.4	antisense	PTCHD3P1	228786_at
ENSG00000205866.2	processed_transcript	FAM99A	
ENSG00000224078.5	processed_transcript	SNHG14	
ENSG00000234636.1	antisense	MED14-AS1	231226_at
ENSG00000179935.5	processed_transcript	LINC00652	220747_at
ENSG00000167459.11	processed_transcript	AC114273.1	206515_at
ENSG00000264247.1	lincRNA	RP11-231E4.4	226924_at

Table S4. External validation in independent samples using the ER positive profile.

	No. of cases/ controls	Agilent probes/ Affy U133A+B probesets	Sensitivity (TP)	Specificity (TN)	Accuracy[a]	P [b]
**ER+ profile (168 lncRNAs)**						
Miller (ln negative, ER positive)	18/106	59/75	94 (17)	1 (1)	48	
Miller/Pawitan (ER positive)	64/260	59/75	91 (58)	25 (66)	58	0.003

[a] Mean balanced accuracy
[b] Fisher's exact test, one-tailed
Abbreviations: TP, true positive; TN, true negative

Table S5. Gene set enrichment analysis (GSEA) identify highly significant pathways associated with top lncRNAs from the overall profile.

GENCODE id	Gene symbol	FDR q-val	Pathways
ENSG00000245532.3	NEAT1	0	RNA_POL_I_PROMOTER_OPENING
		0	PACKAGING_OF_TELOMERE_ENDS
		3.33E-04	AMYLOIDS
		2.50E-04	RNA_POL_I_TRANSCRIPTION
		2.00E-04	DEPOSITION_OF_NEW_CENPA_CONTAINING_NUCLEOSOMES_AT_THE_ CENTROMERE
		3.32E-04	TELOMERE_MAINTENANCE
		2.85E-04	MEIOTIC_RECOMBINATION
		4.99E-04	MEIOTIC_SYNAPSIS
		0.007552	RNA_POL_I_RNA_POL_III_AND_MITOCHONDRIAL_TRANSCRIPTION
ENSG00000240990.5	HOXA11-AS	0	COLLAGEN_FORMATION
		0.007465	EXTRACELLULAR_MATRIX_ORGANIZATION
