# Supplementary material for: Long non-coding RNA expression profiles predict metastasis in lymph node-negative breast cancer independently of traditional prognostic markers
Source: Breast Cancer Res. 2015 Apr 11;17(1):55. doi: 10.1186/s13058-015-0557-4 (PMC4416310; doi:10.1186/s13058-015-0557-4)
Supplement: Additional file 2: Figure S1. — Box plots showing (A) the relative expression levels of the individual long non-coding RNAs (lncRNAs) from the top-10 list of the overall classification in the different subtypes and (B) the relative expression levels of the individual lncRNAs in the independent data sets in the different subtypes. Figure S2 ESR1 gene expression measurements were used to determine estrogen receptor (ER) status. Figure S3 (A) Optimization of the overall classification, resulting in an optimized number of lncRNAs to include in the overall profile (the simplest optimal profile consisted of 47 genes) and (B) optimization of the ER-positive classification, resulting in an optimized number of lncRNAs to include in the ER-positive profile (the simplest optimal profile consisted of 168 genes). We based the mean balanced accuracy on 100 support vector machine (SVM) models in each data point. Figure S4 Expression data matrix of the overall 47-gene profile visualized as a heatmap. Each column represents a tumor sample and each row an lncRNA. Tumor samples are ordered according to their classification probability estimate from 0 to 1. The 47 lncRNAs are order via hierarchical clustering. The metastatic status and ER status are shown in the top bar. Figure S5 Expression data matrix of the ER-positive 168-gene profile visualized as a heatmap. Each column represents a tumor sample and each row an lncRNA. Tumor samples are ordered according to their classification probability estimate from 0 to 1. The 168 lncRNAs are ordered via hierarchical clustering. The metastatic status is shown in the top bar. Figure S6 Kaplan-Meier survival curves of MFS for all validation samples (n = 395) and divided into ER-positive samples (n = 324) and ER-negative samples (n = 71). [file 13058_2015_557_MOESM2_ESM.pdf]

Supplementary Figures

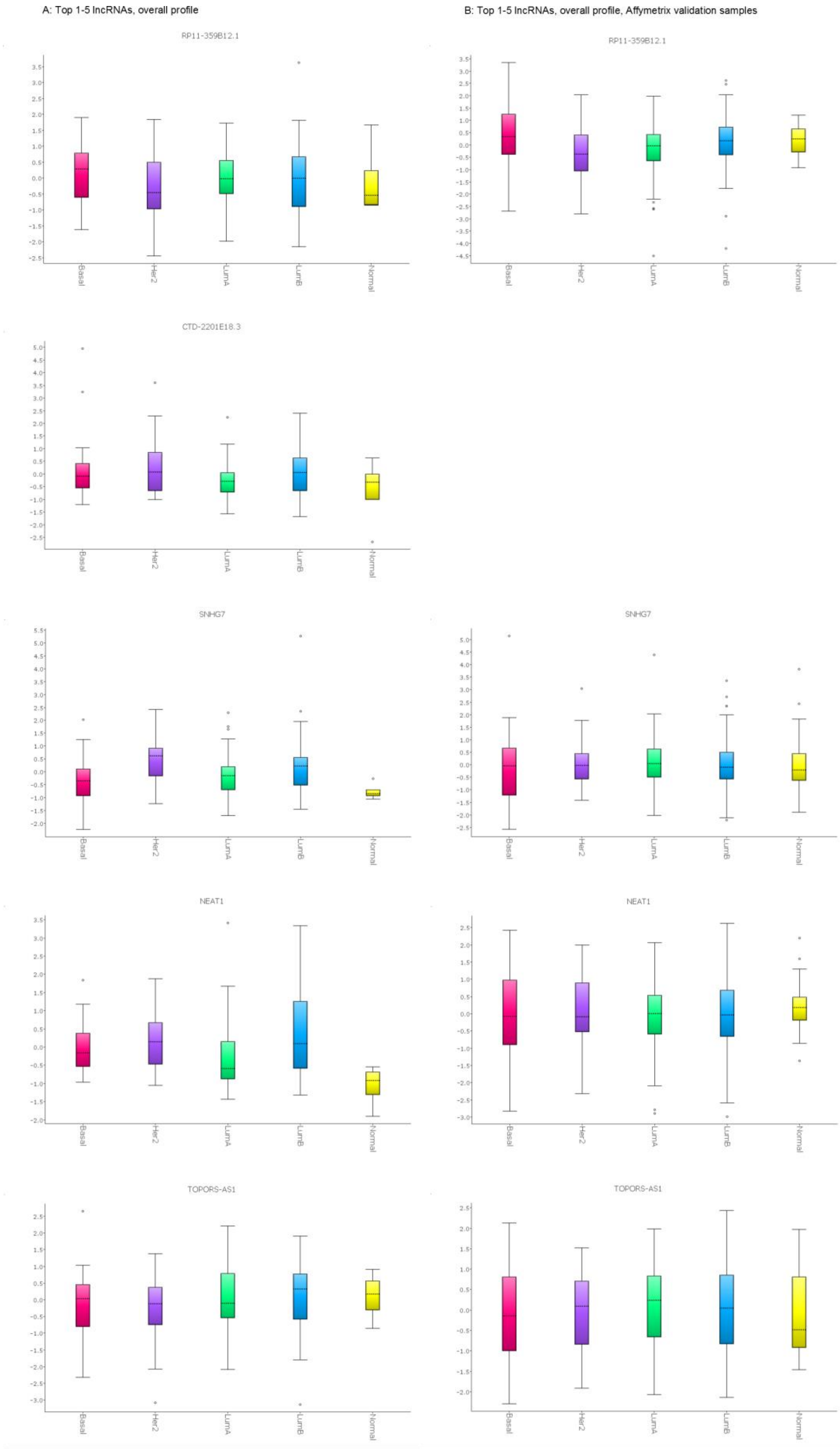

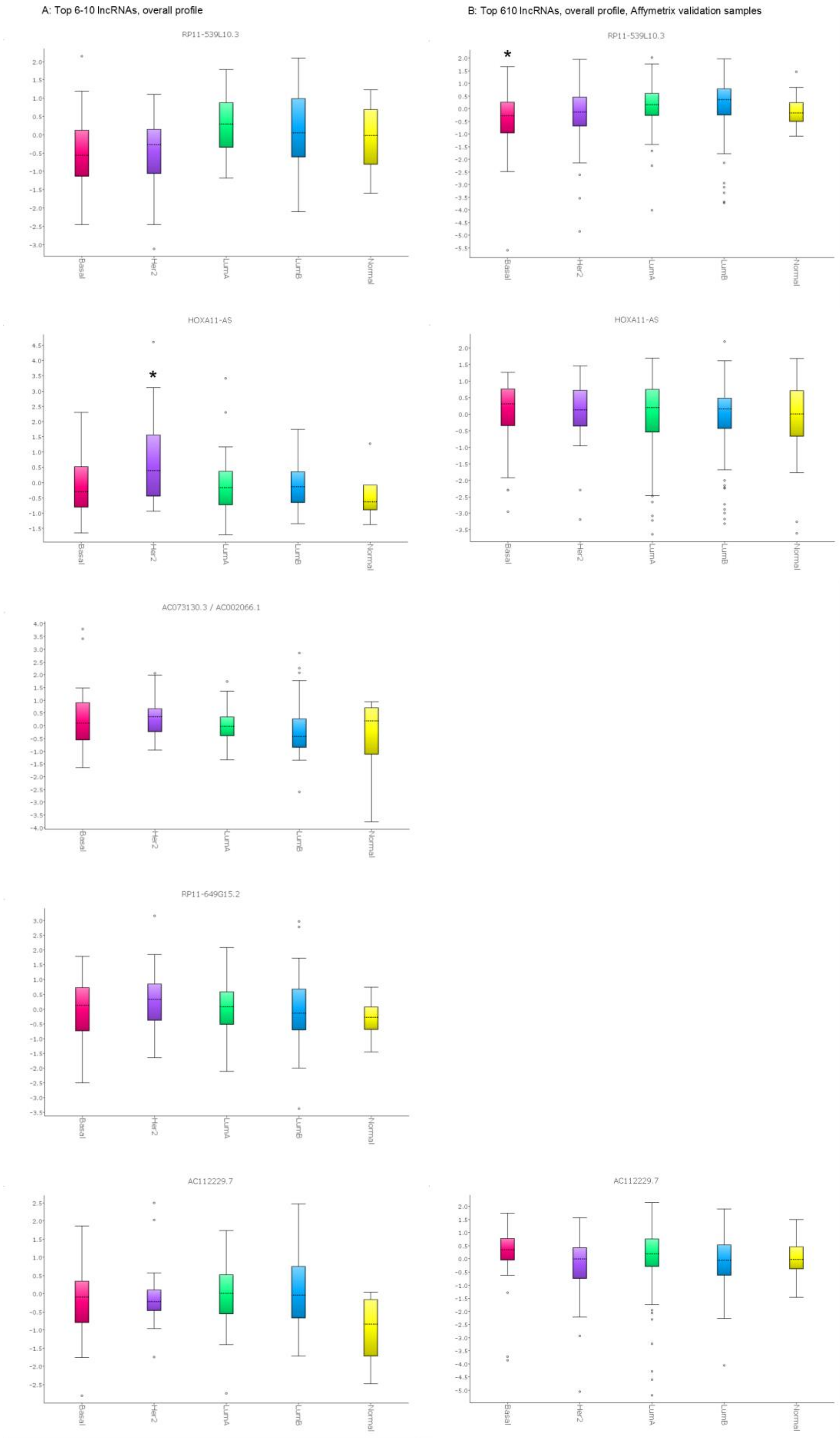

Figure S1. A: Box-plots showing the relative expression levels of the individual lncRNAs, from the top 10 list of the overall classification, in the different subtypes. The expression levels in the subtypes were compared by a t-test; significant associations were reported after adjusting for multiple testing (*= P<0.05). B: Box-plots showing the relative expression levels of the individual lncRNAs, in the independent data sets, in the different subtypes. Seven out of the top 10 lncRNAs were covered by the Affymetrix array. The expression levels in the subtypes were compared by a t-test; significant associations were reported after adjusting for multiple testing (*= P<0.05).

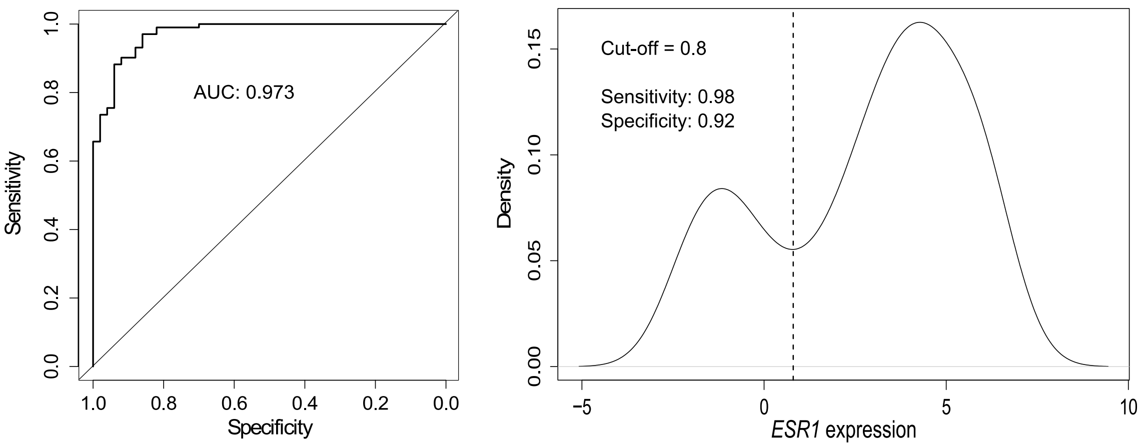

Figure S2. Gene expression measurements of *ESR1* were used to determine ER status. Samples with available immunohistochemical data (n = 152) were used to generate a receiver operating characteristic (ROC) curve with a large area under curve (AUC). The density plot represents the relative expression values of *ESR1* across all samples (n = 164) and determined the cut-off (marked as a dashed line) defining ER status.

A

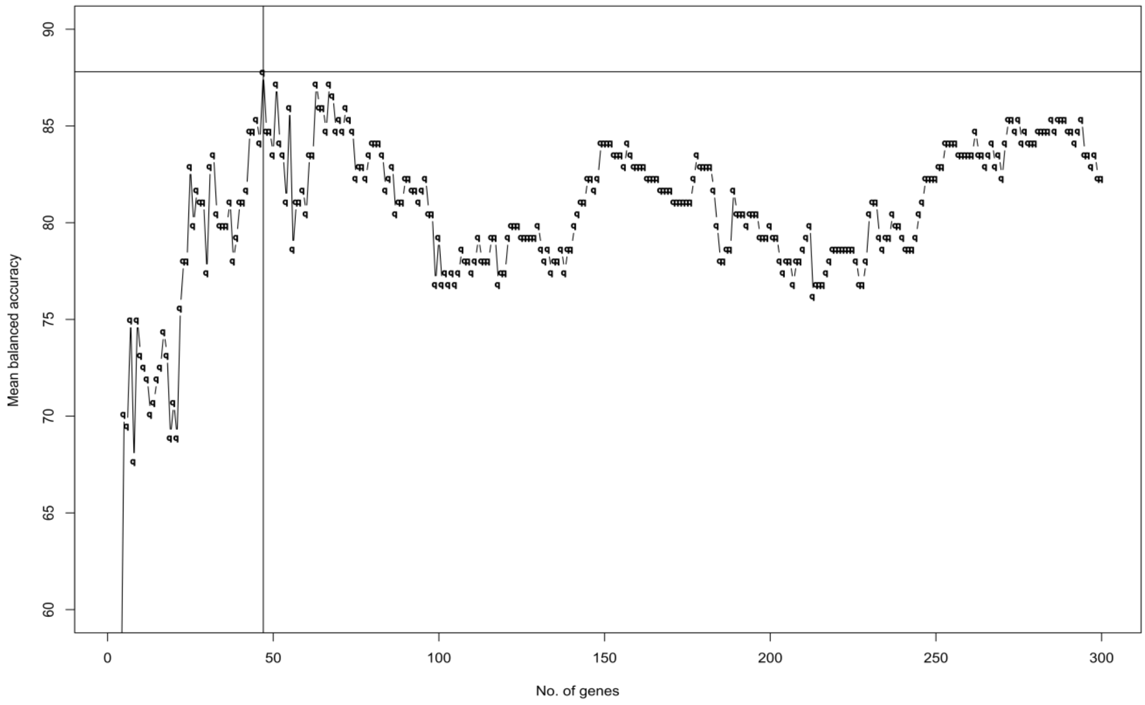

B

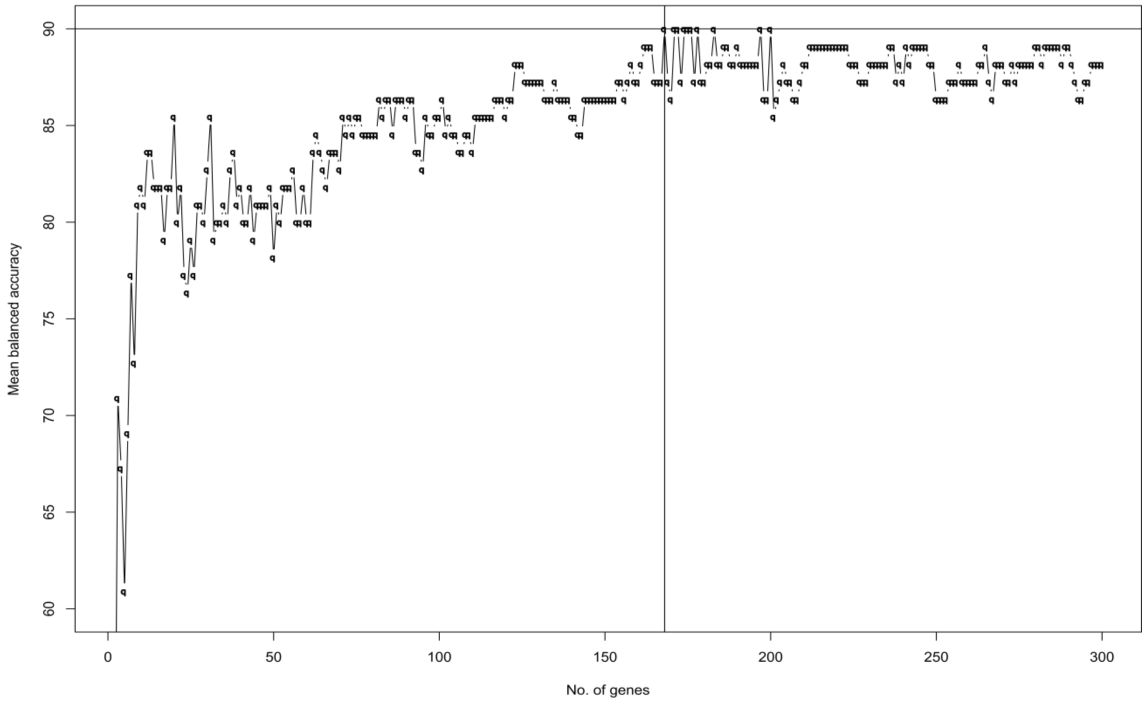

Figure S3. A: Optimization of the overall classification, resulting in an optimized number of lncRNAs to include in the overall profile; the simplest profile consisted of 47 genes.

B: Optimization of the ER+ classification, resulting in an optimized number of lncRNAs to include in the ER positive profile; the simplest profile consisted of 168 genes. We based the mean balanced accuracy on 100 SVM models in each data point.

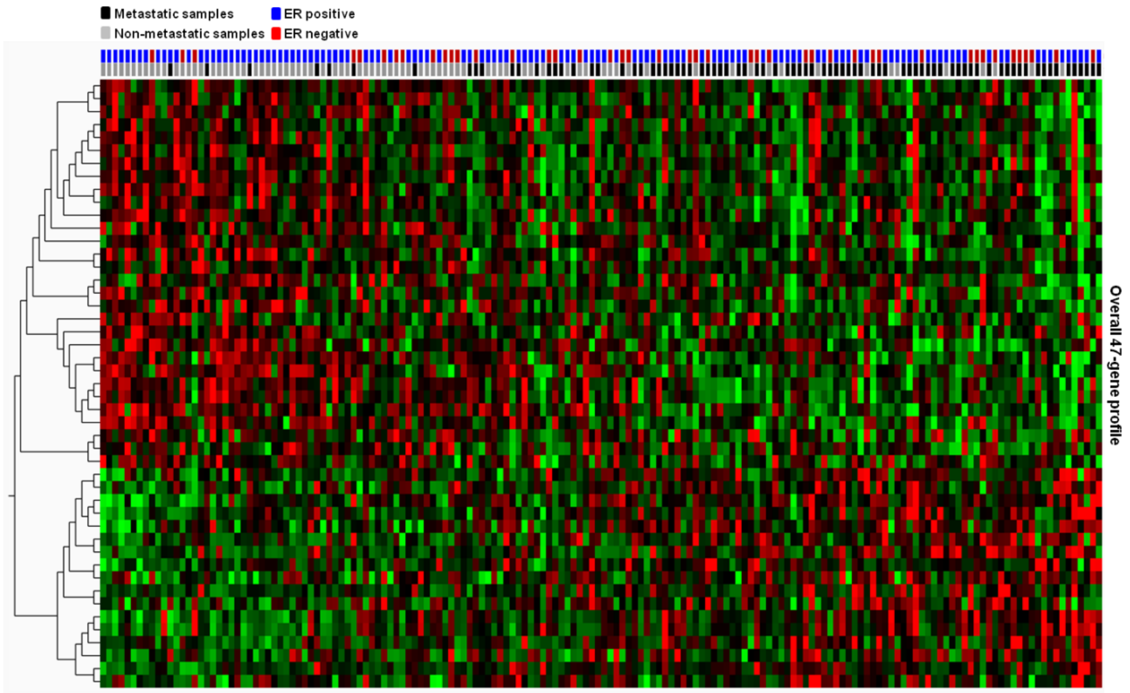

Figure S4. Expression data matrix of the overall 47-gene profile visualized as a heatmap. Each column represents a tumor sample and each row an lncRNA. Tumor samples are ordered according to their classification probability estimate from 0 to 1. The 47 lncRNAs are order via hierarchical clustering. The metastatic status and ER status are shown in the top bar.

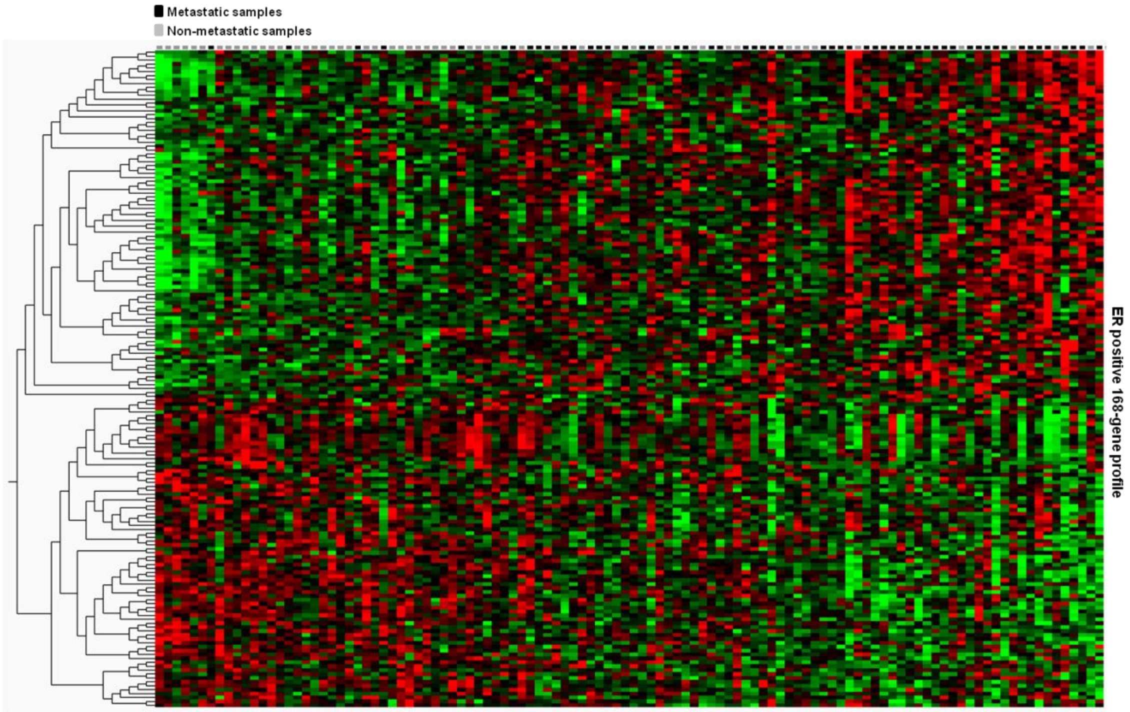

Figure S5. Expression data matrix of the ER positive 168-gene profile visualized as a heatmap. Each column represents a tumor sample and each row an lncRNA. Tumor samples are ordered according to their classification probability estimate from 0 to 1. The 168 lncRNAs are ordered via hierarchical clustering. The metastatic status is shown in the top bar.

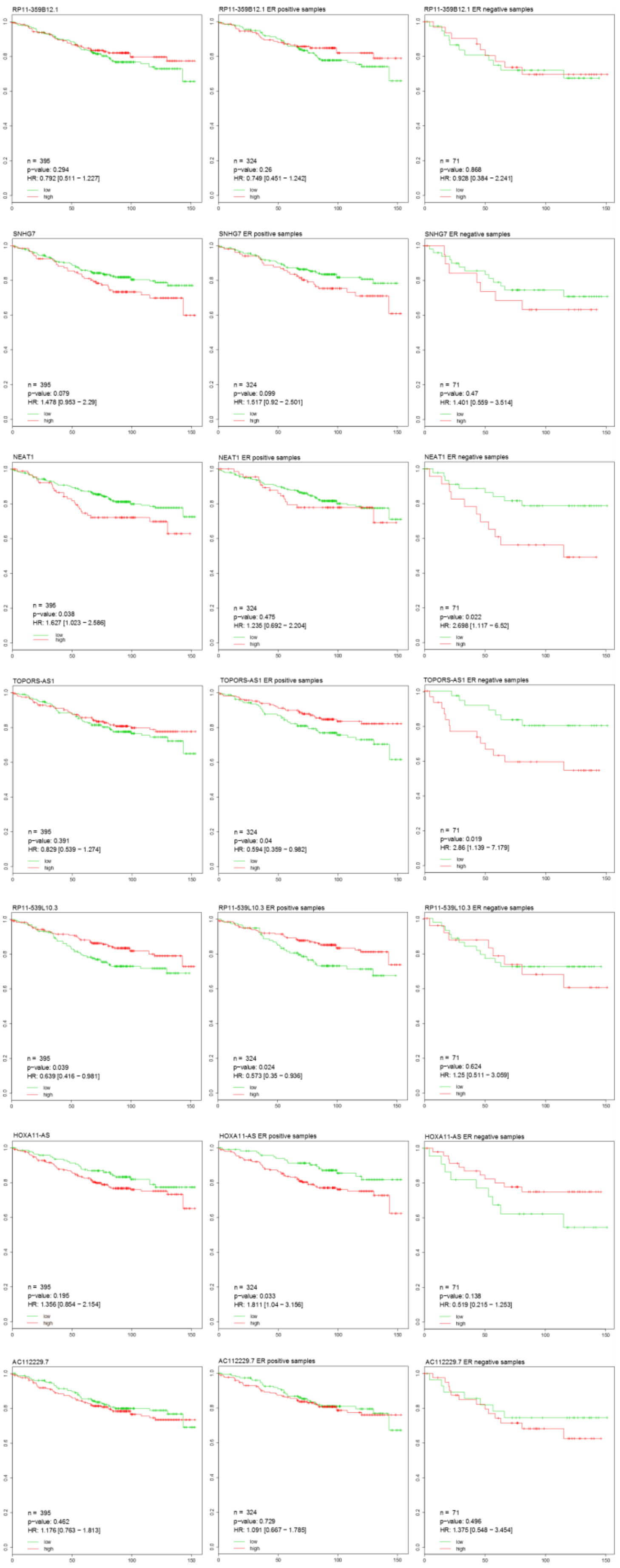

Figure S6. Kaplan-Meier survival curves of MFS for all validation samples (n=395) and divided into ER positive samples (n=324) and ER negative samples (n=71). Plots were used to visualize MFS according to high and low expression of the individual lncRNAs from the top 10 list of the overall classification, 7 out of the top 10 lncRNAs were covered by the Affymetrix array.
